# Clinical and radiologic outcomes in patients with meniscal root tears

**DOI:** 10.1186/s12891-024-07359-4

**Published:** 2024-03-23

**Authors:** Rawee Manatrakul, Maximilian Loeffler, Upasana U. Bharadwaj, Gabby B. Joseph, Drew Lansdown, Brian Feeley, Joe D. Baal, Julio B. Guimaraes, Thomas M. Link

**Affiliations:** 1 Department of Radiology and Biomedical Imaging, University of California San Francisco, 185 Berry St, Suite 350, San Francisco, CA 94158 USA; 2 Department of Radiology, Faculty of Medicine Ramathibodi Hospital, Mahidol University, Bangkok, Thailand; 3 Department of Diagnostic and Interventional Radiology, University Medical Center Freiburg, Freiburg im Breisgau, Germany; 4 Department of Orthopaedic Surgery, University of California San Francisco, San Francisco, CA USA; 5 Department of Radiology, Federal University of Sao Paulo (UNIFESP), Sao Paulo, Brazil; 6 Department of Musculoskeletal Radiology, Fleury Medicina e Saúde, São Paulo, Brazil

**Keywords:** Meniscus, Meniscal root, Treatment, Outcome

## Abstract

**Background:**

Meniscal root tears can lead to early knee osteoarthritis and pain. This study aimed (1) to compare clinical and radiological outcomes between patients who underwent arthroscopic meniscal root repair after meniscal root tears and those who received non-surgical treatment, and (2) to identify whether baseline MRI findings could be potential predictors for future treatment strategies.

**Methods:**

Patients with meniscal root tears were identified from our picture archiving and communication system from 2016 to 2020. Two radiologists reviewed radiographs and MRI studies using Kellgren-Lawrence (KL) grading and a modified Whole Organ MRI Scoring (WORMS) at baseline and follow-up. The median (interquartile range [IQR]) of follow-up radiographs and MRI studies were 134 (44–443) days and 502 (260–1176) days, respectively. MR images were assessed for root tear-related findings. Pain scores using visual analogue scale (VAS) and management strategies (non-surgical vs. arthroscopic root repair) were also collected. Chi-squared tests and independent t-tests were used to assess differences regarding clinical and imaging variables between treatment groups. Logistic regression analyses were performed to evaluate the associations between baseline MRI findings and each future treatment.

**Results:**

Ninety patients were included. VAS pain scores were significantly (*p* < 0.01) lower after arthroscopic repair compared to conservative treatment (1.27±0.38vs.4±0.52) at the last follow-up visit with median (IQR) of 325 (180–1391) days. Increased meniscal extrusion (mm) was associated with higher odds of receiving non-surgical treatment (OR = 1.65, 95%CI 1.02–2.69, *p* = 0.04). The odds of having arthroscopic repair increased by 19% for every 1 mm increase in the distance of the tear from the root attachment (OR = 1.19, 95% CI: 1.05–1.36, *p* < 0.01). The odds of undergoing arthroscopic repair were reduced by 49% for every 1 mm increase in the extent of meniscal extrusion (OR = 0.51, 95% CI: 0.29–0.91, *p* = 0.02) as observed in the baseline MRI.

**Conclusions:**

Patients who underwent arthroscopic repair had lower pain scores than patients with conservative treatment in the follow-up. Distance of the torn meniscus to the root attachment and the extent of meniscal extrusion were significant predictors for arthroscopic repair in the next three weeks (time from the baseline MRI to the surgery date).

## Introduction

Meniscal root tears may occur due to acute knee injury typically in the younger population, or due to degenerative processes mostly in older patients, in particular overweight women [1]. In an acute setting, meniscal root tears are reported to be related to hyperflexion or squatting mechanisms [2]. The posterior root of the medial meniscus is the most common location of meniscal root tears because it has reduced mobility and takes most of the force applied to the medial compartment [2, 3]. A meniscal root tear increases peak contact pressure in the medial compartment of the knee and decreases the contact area [4]. Meniscal root injury leads to loss of hoop stress resistance, meniscal extrusion, and early degenerative change of the knee, especially in the medial compartment [4]. Also noted is a significant correlation between injury of the posterior root of the medial meniscus and insufficiency fractures at the medial joint compartment [5–8]. The widely accepted definition of a meniscal root tear is a bony and/or soft tissue avulsion within 1 cm from the meniscal root attachment [9]. Diagnosis of meniscal root injury based on clinical and radiographic findings may be challenging, and magnetic resonance imaging (MRI) is the standard imaging test [9]. Historically, treatment of meniscal root injury was focused on partial meniscectomy [9], but due to the importance of the meniscal root as described, meniscal root repair techniques are more frequently used to restore the native function, anatomy, and biomechanics of the meniscal root attachment [2, 9, 10]. While studies have investigated surgical techniques and outcomes, there is limited information on imaging and clinical outcomes of conservative treatment in direct comparison with surgical intervention and on baseline MR imaging variables which predict future arthroscopic meniscal root repair [11, 12].

Our study therefore focused on the relationship between clinical data, imaging findings, and management strategies in patients with meniscal root tears. Our study aimed to compare clinical and radiological outcomes between patients who underwent arthroscopic meniscal root repair after meniscal root tears and those who received non-surgical treatment. In addition, we aimed to identify whether baseline MRI findings could be potential predictors for future treatment strategies, including non-surgical treatment and, arthroscopic repair.

## Materials and methods

### Patient cohort and selection criteria

Radiology reports in the institutional picture archiving and communication system (PACS) database were searched for “meniscal root tear” in a 5-year period from 2016 to 2020 using the search engine mPower (Nuance, Burlington, MA, USA). Inclusion criteria were patients aged 20–70 with degenerative lesions, partial tears, or complete tears of the meniscal root based on a consensus review of MRI studies (JBG, TML) as well as available follow-up visits. Using additional exclusion criteria including a history of knee surgery, significant knee deformity due to knee injury, inflammatory arthropathies, and local malignant tumors, 90 patients were identified for this study. Note that concomitant injuries of the other ligaments of the knee were not our exclusion criteria. The study was HIPAA compliant and approved by the institutional review board.

Regarding follow-up visits, we collected the clinical data at the visits after the treatment (i.e., the outpatient visits after the arthroscopic meniscal root repair, the visit after the physical therapy). Clinical data with associated treatment follow-up were reviewed with one orthopedic surgeon (DL). For the follow-up radiographs, we selected one follow-up radiograph per patient for further analysis using the following method: First, we calculated the median follow-up time (days) from all patients’ first follow-up and baseline radiographs. Then, we selected the radiograph with the closest time to follow-up compared to the calculated median (134 days) for each patient to be the follow-up radiograph. For the follow-up MRI, if patients had more than one follow-up MRI, we used the MRI study covering the largest time interval between surgery and follow-up imaging for the analysis.

### Demographic and clinical data

The electronic medical records were retrospectively reviewed to collect the following data: baseline patient characteristics (sex, age, body mass index [BMI]), date of the first visit with symptoms related to meniscal root tear, duration of symptoms, side of the symptomatic knee, visual analogue scale (VAS) pain scores at the first and follow-up visits, treatment received including the date of surgery. We divided the treatment groups into non-surgical treatment and arthroscopic repair for the analyses. Non-surgical treatment included pharmacotherapy and physiotherapy.

### Imaging data

#### Radiographic acquisition

Standardized weight-bearing posteroanterior knee radiographs were obtained in a fixed flexion position of the symptomatic knee in all patients with 20°–30° flexion and 10° external rotation of the feet positioning. Radiographs were obtained at baseline, when patients presented with symptoms, and after surgery or during follow-up when patients were managed conservatively as indicated by clinical symptoms.

#### MRI acquisition

Each patient had at least a single MRI of the symptomatic knee using a 3T GE Excite Signa MRI Scanner (General Electric, Milwaukee, WI, USA) with a dedicated 8-channel transmit-receive phased-array knee coil (Invivo, Orlando, FL, USA). The following MR sequences and parameters were used: sagittal proton density-weighted (PD) fast spin echo (FSE) sequence (repetition time (TR)/echo time (TE), 2500–4000 ms/ 20–30 ms; 3 mm slice thickness; 0 mm interslice gap), sagittal, coronal, and axial intermediate weighted FSE sequences with fat suppression (TR/TE, 4000–6000 ms/ 40–60 ms; 3 mm slice thickness; 0 mm interslice gap), and a coronal T1-weighted FSE sequence (TR/TE, 700–900 ms/ 5–10 ms; 3 mm section thickness; 0 mm interslice gap). Voxel size was similar for all sequences used and depended on joint size and required field of view for imaging of the knee, it varied between 0.27 and 0.31 × 0.27–0.31 × 3–4 mm^3^.

### Image analysis

All imaging studies were interpreted independently by two board-certified musculoskeletal radiologists (TML, JBG) with 25 years and 10 years of experience, respectively. Adjudication readings were performed in case of discordant or equivocal findings by both radiologists. Both radiologists were blinded to the clinical information of all patients.

All knee radiographs at baseline and follow-up time points were graded according to the Kellgren-Lawrence (KL) classification by the two board-certified musculoskeletal radiologists as above [13]. The KL classification is briefly described as grade 0 (no osteoarthritis), grade 1 (doubtful joint space narrowing or doubtful osteophyte), grade 2 (possible/mild joint space narrowing and definite osteophytes ), grade 3 (definite/moderate joint space narrowing with moderate sized osteophytes) and grade 4 (complete loss of joint space with osteophytes). Follow-up radiographs were typically obtained at the same time as the clinical follow-up visits and clinical and radiographic data were collected at this time.

**Fig. 1:**
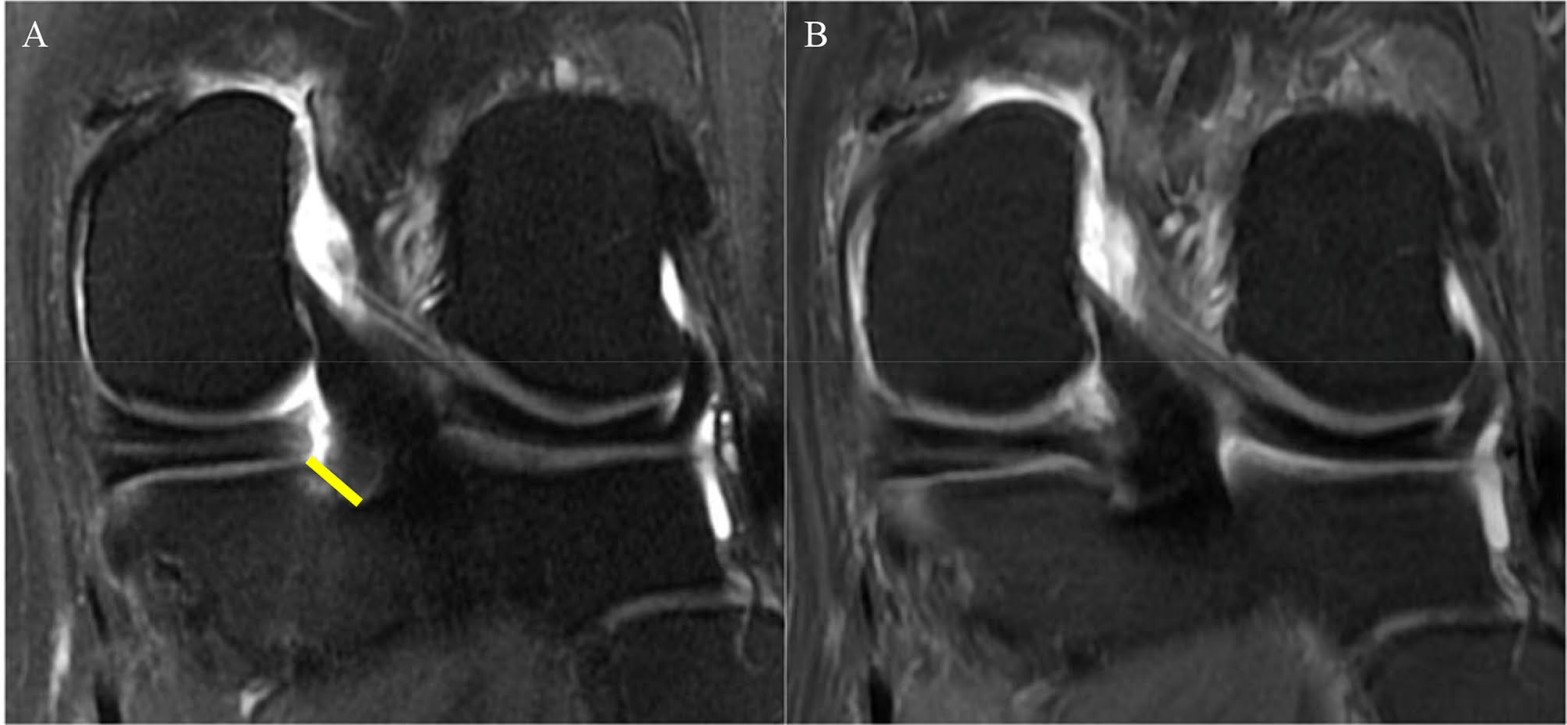
A 58-year-old woman presented with right knee pain and swelling for a month. (A) Baseline MRI showed a radial tear at the posterior root of the medial meniscus. The distance from the torn meniscal root to its bony attachment was 8 mm (yellow line). Interestingly no significant meniscal extrusion was noted at this time. (B) At 7 months after the arthroscopic meniscal root repair. The meniscal root showed complete healing. Note the slightly increased signal intensity in the repaired meniscal root, which is a normal post-operative finding [17]

**Fig. 2:**
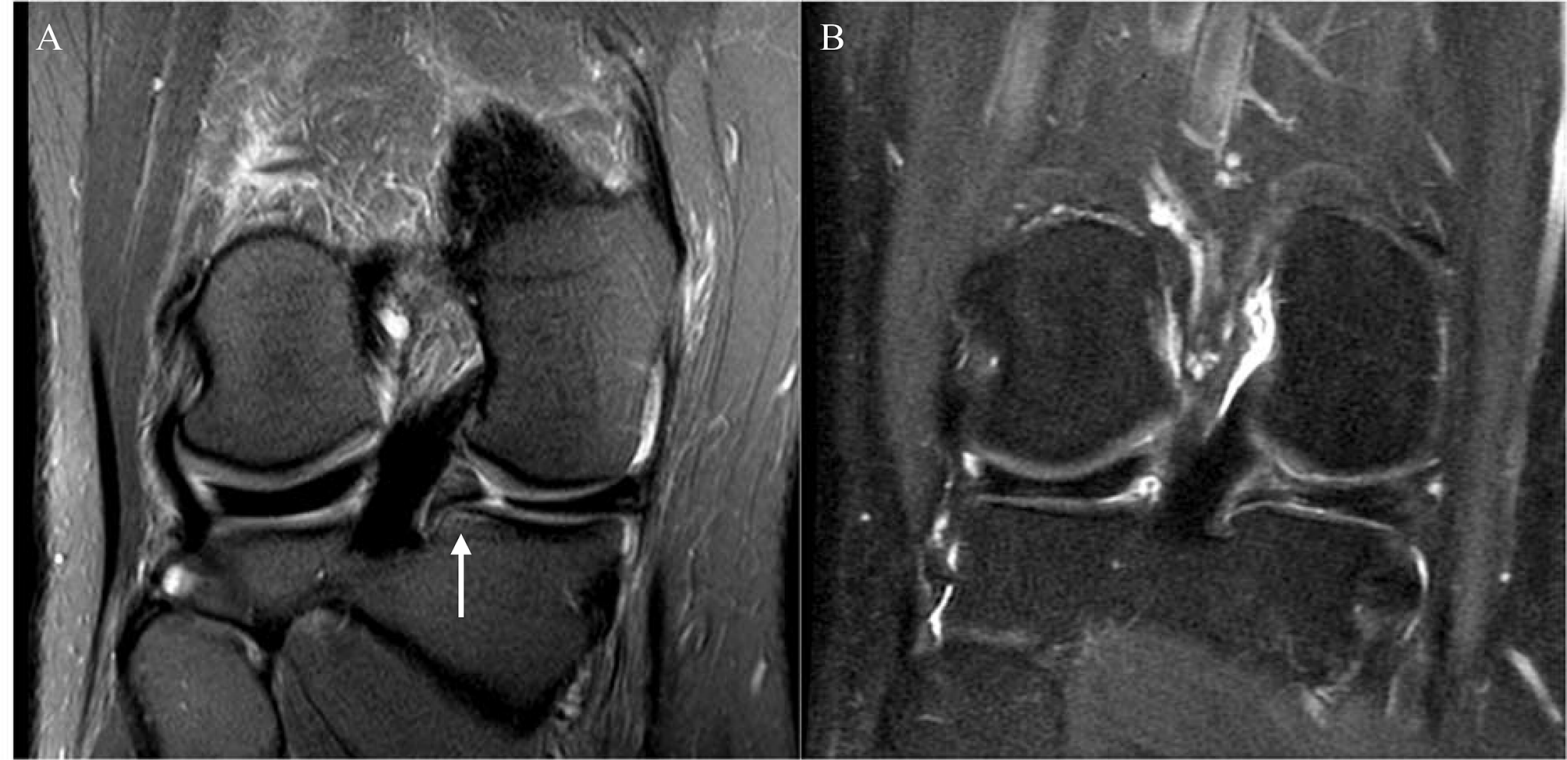
A 45-year-old woman presented with left knee pain and swelling for three days. (A) Baseline MRI showed a radial tear at the posterior root of the medial meniscus with associated bone marrow edema-like lesion at the meniscal root attachment (arrow). (B) At three years after the arthroscopic meniscal root repair. The meniscal root showed partial healing with no longer seen associated bone marrow edema-like lesion

All MRI studies were assessed for meniscal tear-related findings, including the distance of torn meniscal roots from their attachment (mm) [10] (Fig. 1), the extent of meniscal extrusion (mm) [14, 15], bone marrow edema-like lesions (BMELs), subchondral cysts, and the presence of regional synovitis at the meniscal root attachment (Fig. 2), as well as the presence of a subchondral bone fracture. We also assessed the structural degenerative disease burden of the entire knee using the semiquantitative Whole Organ MRI Score (WORMS) grading system [16]. We binarized WORMS for cartilage as no cartilage defect (WORMS ≤ 1) and presence of cartilage defects (WORMS ≥ 2). We also binarized WORMS for BMELs as no BMEL (WORMS = 0) and the presence of BMELs (WORMS ≥ 1). The data regarding WORMS of cartilage and bone marrow abnormalities were collected separately for six knee regions (patella, trochlea, medial femoral condyle, lateral femoral condyle, medial tibia, and lateral tibia).

To define complete and partial healing as well as complete retear in the MRI images we used the method previously described by Guimarães [17] et al.: continuity of the repaired root in the axial, coronal and sagittal planes was defined as complete healing, continuity of the repaired root in one or two planes was assessed as partial healing, and no continuation of the repaired root in all three planes was defined as a complete retear.Two radiologists performed inter- and intra-reader reproducibility assessments of the torn meniscal root findings on MRI (TL, JBG). Inter-reader intra-class correlation coefficients (ICCs) and intra-reader ICCs were calculated and reported in a previous study [18]. ICCs for inter-reader agreement ranged from 0.80 to 0.84, and intra-reader agreement ranged from 0.86 to 0.88.

### Statistical analysis

Statistical analyses were performed using Stata Statistical Software (version 18.0, College Station, TX: Stata Corp LP). Descriptive statistics for patient characteristics, imaging findings, duration of the follow-up, and clinical outcomes were tabulated in each treatment group (non-surgical and arthroscopic repair). The time (days) from baseline MRI to arthroscopic repair was also calculated and reported in median (interquartile range [IQR]).

Chi-squared tests were used to assess differences in categorical variables (i.e., sex, presence of meniscal extrusion, cartilage defect, BMEL, and subchondral fracture) between treatment groups, while independent t-tests were used to assess the differences in continuous variables (i.e., age, BMI, VAS pain score, duration of symptoms, the distance of tear to the bony attachment, and extent of meniscal extrusion) between treatment groups at baseline time point.

Linear regression analyses were used to evaluate the associations between treatment group (predictor) and VAS pain score (outcome) at each follow-up time point. These analyses were performed with and without adjustment for the duration to follow-up (time in days).

Logistic regression analyses were utilized to evaluate the associations between baseline MRI findings (i.e., presence of insufficiency fracture, BMEL, cartilage defect, and the distance of the torn roots to their attachment [mm]) and each future treatment, including getting non-surgical treatment (yes/no) and arthroscopic repair (yes/no). All analyses were adjusted for age, sex, and BMI. A P-value of < 0.05 was considered statistically significant.

## Results

### Clinical follow-up

All patients analyzed in this study (*n* = 90) had at least one follow-up visit after the treatment. All of the patients had lesions at the posterior root, and 6 patients had lesions at the posterior root of the lateral meniscus. Forty-nine (54.44%) patients had three or more follow-up visits and 11 (12.22%) patients had two follow-up visits. The median (IQR) time to the first, second, and last follow-up visit was 33 (15–72), 57 (49–96), and 325 (180–1391) days, respectively. VAS pain scores were available for 90, 79, 55, and 46 patients at the baseline, first, second, and last follow-up visits, respectively. Fifty-five (61.11%) patients were treated non-surgically and 35 (38.89%) patients underwent arthroscopic meniscal root repair using a standard transtibial pull-out meniscal root repair with 2 luggage tag sutures at the posterior horn of the meniscus and fixation with a cortical button and/or bioabsorbable anchors, as previously described by Woodmass and Krych et al. [19].

The participant characteristics are listed in Table 1. At baseline, there was no significant difference in the distribution of males/females (*p* = 0.11), mean BMI (*p* = 0.58), or mean VAS pain score (*p* = 0.73) between the non-surgical and arthroscopic repair groups. Age significantly differed between groups (*p* < 0.01) with lower average age in the arthroscopic repair patients than in the non-surgical patients (51.46±10.45 vs. 57.25±7.81 years).

**Table 1:** Baseline characteristics and imaging findings

Variable	Non-surgical treatment (*N* = 55)	Arthroscopic repair (*N* = 35)	P-value
**Sex (year), N (%)**			
Female	43 (78.18)	22 (62.86)	
Male	12 (21.82)	13 (37.14)	0.11
Age (year), mean (±SD)	57.25 (7.81)	51.46 (10.45)	< 0.01*
BMI (kg/m^2^), mean (±SD)	29.63 (6.47)	28.90 (5.15)	0.58
**Side of the symptomatic knee, N (%)**			
Left	30 (54.55)	21 (60)	
Right	25 (45.45)	14 (40)	0.61
Pain score (VAS) at the first visit, mean (±SD)	5.6 (2.07)	5.43 (2.54)	0.73
Duration of symptoms (day), mean (±SD)	132 (275)	90 (98)	0.39
**Baseline radiographic findings**			
KL, mean (±SD)	0.91 (1.01)	0.91 (0.78)	0.99
**Baseline MRI findings**			
Distance of tear from root attachment (mm), mean (±SD)	4.02 (3.2)	6.43 (5.21)	< 0.01*
Meniscal extrusion, N (%)	45 (81.82)	24 (68.57)	0.15
Extent of extrusion (mm), mean (±SD)	4.33 (1.24)	3.51 (1.02)	< 0.01*
**WORMS for cartilage, N (%)**			
0–1	7 (12.73)	6 (17.14)	
≥ 2	48 (87.27)	29 (82.86)	0.56
BMEL at the torn meniscal root attachment, N (%)	17 (30.91)	18 (51.43)	0.05
BMEL at the medial femoral condyle, N (%)	17 (30.91)	10 (28.57)	0.81
BMEL at the lateral femoral condyle, N (%)	1 (1.82)	0	0.42
BMEL at the medial tibia, N (%)	12 (21.82)	9 (25.71)	0.67
BMEL at the lateral tibia, N (%)	3 (5.45)	1 (2.86)	0.56
BMEL at the patella, N (%)	16 (29.09)	11 (31.43)	0.81
BMEL at the trochlear, N (%)	20 (36.36)	8 (22.86)	0.18
Subchondral bone fracture, N (%)	10 (18.18)	3 (8.57)	0.21

BMI, body mass index; VAS, visual analogue scale; KL, Kellgren-Lawrence; WORMS, Whole Organ MR Scoring; BMEL, bone marrow edema-like lesions

Patients with arthroscopic repair had significantly lower VAS pain scores compared to those with non-surgical treatment at the first, second, and last follow-up visit. The results were consistent after adjustment for time to follow-up (Table 2).

**Table 2:** VAS pain scores at the follow-up visits

	Time to follow-up (days) Median (IQR)	VAS pain score unadjusted				VAS pain score adjusted for time to follow-up			
		Mean (±SD)	Coefficient (SE) compared to reference	95%CI	P value compared to reference	Adjusted Mean (±SD)	Coefficient (SE) compared to reference	95%CI	P value compared to reference
First follow-up (*n* = 79)	33 (15–72)								
Non-surgical (*n* = 45)	45 (19–102)	4.16 (0.38)		reference		4.07 (0.39)		reference	
Arthroscopic repair (*n* = 34)	21 (14–34)	2.82 (0.44)	-1.33 (0.58)	-2.48, -0.18	0.02*	2.93 (0.45)	-1.14 (0.60)	-2.34, 0.06	0.06
Second follow-up (*n* = 55)	57 (49–96)								
Non-surgical (*n* = 21)	75 (53–301)	3.14 (0.45)		reference		2.80 (0.48)		reference	
Arthroscopic repair (*n* = 34)	53 (49–70)	1.32 (0.35)	-1.82 (0.57)	-2.96, -0.68	< 0.01*	1.47 (0.36)	-1.33 (0.63)	-2.59, -0.07	0.04*
Last follow-up (*n* = 46)	325 (180–1391)								
Non-surgical (*n* = 16)	1514 (331–1884)	4.00 (0.52)		reference		3.95 (0.59)		reference	
Arthroscopic repair (*n* = 30)	241 (152–419)	1.27 (0.38)	-2.73 (0.64)	-4.02, -1.44	< 0.01*	1.32 (0.40)	-2.63 (0.76)	-4.15, -1.10	< 0.01*

VAS, visual analogue scale; 95%CI, 95% confidence interval

### Radiographic findings at baseline and follow-up

There was no significant difference in mean KL grade at baseline between non-surgical and arthroscopic repair patients (*p* = 0.99), Table 1. A follow-up knee radiograph was available in 49 patients with a median (IQR) time to follow-up of 134 (44–443) days. There was a significantly lower (*p* < 0.01) mean KL grade (±SD) in the arthroscopic repair group 1.04±0.84) compared to the non-surgical group (2.04±0.96), with a mean difference (±SD) of 1.00 (±0.26) as shown in Table 3.

**Table 3:** Kellgren-Lawrence grades in a subset of patients where both baseline and follow-up radiographs were available

Variable	Time to follow-up (days) Median (IQR)	Kellgren-Lawrence grade			
		Mean (±SD)	Δ Mean (±SD) compared to reference	95%CI	P value compared to reference
**Baseline (** *n* ** = 49)**					
Non-surgical(*n* = 24)		1.17 (1.05)		reference	
Arthroscopic repair (*n* = 25)		0.84 (0.75)	0.33 (0.26)	-0.19-0.85	0.21
**Follow-up (** ***n*** ** = 49)**	134 (44–443)				
Non-surgical (*n* = 24)	399 (151–907)	2.04 (0.96)		reference	
Arthroscopic repair (*n* = 25)	52 (27–134)	1.04 (0.84)	1.00 (0.26)	0.49–1.52	< 0.01*

95%CI, 95% confidence interval

### MRI findings at baseline and follow-up

At baseline, interestingly there was relatively limited meniscal extrusion in the entire patient cohort and there was even less extent of meniscal extrusion (3.51±1.02 vs. 4.33±1.24 mm, *p* < 0.01) in the arthroscopic repair group at baseline compared to the non-surgical group. There was a significantly greater distance of the torn meniscal root from the bony attachment (6.43±5.21 vs. 4.02±3.2 mm, *p* < 0.01) in the arthroscopic repair group at baseline compared to the non-surgical group. However, there was no significant difference in the presence of meniscal extrusion, cartilage defects or BMELs in the baseline MRI studies as shown in Table 1.

A follow-up MRI was available in 16 patients with a median (IQR) time to follow-up of 502 (260–1176) days. Follow-up MRI studies were available in 6 patients after non-surgical treatment and in 10 patients with arthroscopic repair. In 1/6 of the non-surgical treatment patients no change of the meniscal root tear was shown, with a decreased gap in 2/6 and an increased gap in 3/6 patients. In the arthroscopic repair patients, complete healing of the meniscal root tear was found in 3/10 patients (Fig. 1), partial healing in 3/10 patients (Fig. 2), and meniscal root complete retear after the arthroscopic repair in 4/10 patients.

### Baseline MRI findings as predictors for non-surgical treatment

Increased meniscal extrusion (mm) was associated with higher odds of receiving non-surgical treatment (OR = 1.65, 95%CI 1.02–2.69, *p* = 0.04). Increased distance of the torn meniscal root from the bony attachment (mm) was associated with lower odds of receiving non-surgical treatment (OR = 0.87, 95%CI 0.77–0.98, *p* = 0.02). The presence of BMEL at the torn meniscal root was associated with lower odds of receiving non-surgical treatment (OR = 0.31, 95%CI 0.12–0.76, *p* = 0.01). The details of the logistic regression analyses for non-surgical treatment outcome are listed in Table 4.

**Table 4:** Baseline MRI findings as predictors for each future treatment

	OR	95%CI	P-value
**Dependent: Non-surgical treatment**			
Distance of tear to root attachment (mm)	0.87	0.77–0.98	0.02*
BMEL at the torn meniscal root attachment (yes/no)	0.31	0.12–0.76	0.01*
Extent of meniscal extrusion (mm)	1.65	1.02–2.69	0.04*
Cartilage defect at the lateral tibia (yes/no)	3.03	0.89–10.33	0.08
**Dependent: Arthroscopic repair**			
Distance of tear to root attachment (mm)	1.19	1.05–1.36	< 0.01*
Extent of meniscal extrusion (mm)	0.51	0.29–0.91	0.02*
Subchondral bone fracture (yes/no)	0.43	0.11–1.75	0.24
Cartilage defect at the lateral tibia (yes/no)	0.35	0.09–1.38	0.13

All analyses were adjusted for age, sex, and BMI at baseline. OR, odds ratio; 95%CI, 95% confidence interval; BMEL, bone marrow edema-like lesion

### Baseline MRI findings as predictors for arthroscopic repair

Increased distance of the torn meniscal root from the bony attachment (mm) was associated with higher odds of undergoing arthroscopic repair (OR = 1.19, 95%CI 1.05–1.36, *p* < 0.01). Greater extent of meniscal extrusion (mm) was associated with lower odds of undergoing arthroscopic repair (OR = 0.51, 95%CI 0.29–0.91, *p* = 0.02). The median (IQR) time from baseline MRI to arthroscopic repair was 22 (14–34) days. The details of the logistic regression analyses for arthroscopic repair outcome are listed in Table 4.

### Sensitivity analysis

Due to wide variability in timing of the post treatment visits we also performed a linear regression analysis using the treatment groups as predictors and using VAS pain score at the follow-up visits as outcomes with and without adjustment for time to follow-up (days), the adjusted models showed a possible trend at the first follow-up visit (*p* = 0.06) and were significant for the other follow-up visits (*p* < 0.05). Performing a sensitivity analysis and excluding the root tears of the lateral meniscus, we found similar results with the exception of some of the MRI predictors for future treatment; BMEL at the torn meniscal root attachment and extent of meniscal extrusion showed a possible trend (*P* = 0.05 resp. 0.06) for conservative treatment and extent of meniscal extrusion showed a possible trend (*P* = 0.06) for surgical treatment.

## Discussion

Our study showed that patients who underwent arthroscopic meniscal root repair had significantly lower VAS pain scores after treatment compared to the non-surgical groups at the median follow-up time of three weeks, seven weeks, and eight months. Patients with more severe meniscal extrusion at baseline tended to have non-surgical treatment and were less likely to undergo arthroscopic repair in the future. At the follow-up visit, a significantly lower mean KL grade was observed in the arthroscopic repair group. Our study also revealed a higher chance of undergoing arthroscopic repair with a larger distance of torn meniscal roots from the bony attachment.

A previous study by Krych et al. [20] showed that patients who underwent meniscal root repair had significantly improved VAS pain scores (2.8 vs. 0.7, *p* < 0.01) two years after surgery, which is similar to our study. This is likely because the arthroscopic repair restores the function of the meniscus as the shock absorber, as shown in a previous human cadaveric study [4]. One potential explanation of the association between the extent of meniscal extrusion and the odds of receiving non-surgical treatment is that meniscal extrusions may be related to lower meniscal quality and more tearing – potentially, orthopedic surgeons did not recommend surgery because the meniscus is too degenerated. Falkowski et al. [21] reported evidence supporting this hypothesis that the extent of meniscal extrusion was larger when the meniscus had more mucoid degeneration compared to a normal meniscus.

A previously published systematic review [22] showed some degree of KL grade progression at the final follow-up time point regardless of the treatment strategy. However, there was faster progression in non-surgical and meniscal debridement groups compared to the arthroscopic repair groups. Despite the relatively short follow-up time for radiographs in our study, our results showed a similar result compared to previous studies with a longer follow-up duration. The mean KL grade of our non-surgical group changed from 1.17 to 2.04 at the 13-month follow-up, while Krych et al. [23] showed grades of 1.5 to 2.4 at the 62-month follow-up and Neogi et al. [24] showed grades of 1 to 1.8 at the 35-month follow-up.

Scher et al. showed that knee osteoarthritis patients with BMELs were more likely to have disease progression and were more likely to have total knee arthroplasty within three years (OR = 8.95, *p* = 0.02) [25]. This may be explained by the fact that BMELs have been related to more advanced pain and structural degeneration [26–28]. Tanamas et al. [29] also reported that the presence and severity of BMELs in patients with symptomatic knees were associated with greater cartilage loss at 2-year MRI follow-up. Our study suggests that BMELs at the torn meniscal root attachment are associated with or mediate the effect of pain, arthritis, and cartilage loss, resulting in a lesser likelihood of getting only the non-surgical treatment in the future. However, further studies with larger sample sizes and analysis of mediating factors are needed to better understand these complex relationships.

While many studies investigated the association between baseline imaging variables and the odds of future knee arthroplasty, to the best of our knowledge no study has investigated the association between baseline imaging parameters and the odds of future arthroscopic meniscal root repair. Our study showed a higher chance of undergoing arthroscopic repair with a larger distance of torn meniscal roots from their bony attachment. We investigated this parameter because previous studies had already shown the association between this parameter and peak contact pressure [4, 30]. LaPrade et al. [30] conducted a human cadaveric study revealing that contact pressure within the lateral knee compartment increased when the distance of the torn lateral meniscal root from the bony attachment site increased. We hypothesize that more pressure in the knee joint may be related to more pain via local nociceptors [31, 32] and may affect the patient’s decision to undergo arthroscopic repair.

The discrepancy of increased likelihood of arthroscopic meniscal root repair with larger distance of torn meniscal roots from their bony attachment and decreased likelihood of repair with increased meniscal extrusion may be due to more advanced degeneration of the meniscus related to extrusion and presence of high grade cartilage lesions or other significant degenerative pathology in the same compartment of the torn meniscal root are contraindication for the meniscal root repair [33, 34]. Our results supported this hypothesis by showing that there were more cartilage defects (WORMS cartilage ≥ 2) in patients with non-surgical treatment at the baseline MRI (87.27% vs. 82.86%, *p* = 0.56), but without statistical significance, probably due to the low number of subjects in our cohort. Interestingly, our study revealed that patients with arthroscopic repair had a lower average age compared to the non-surgical group. This may be explained by the fact that patients at younger age are physically more active and have overall less structural degeneration of the knee.

Our study has some limitations: given the retrospective study design there was limited data availability, and loss of follow-up with only 54.44% of our cohort having three or more follow-up visits. There was also more heterogeneity of the follow-up protocol regarding both clinical and imaging aspects which resulted in high standard deviations and interquartile ranges of our data.

## Conclusions

Patients who underwent arthroscopic repair for meniscal root tears had significantly lower pain and less progression of radiographic knee OA at follow-up compared to patients who did not undergo surgery. The distance between torn meniscal root and bony attachment was associated with higher odds to undergo arthroscopic meniscal root repair, whereas the extent of meniscal extrusion was associated with a lower likelihood of undergoing arthroscopic repair.

## Data Availability

The data that support the findings of this study are not openly available due to reasons of patient privacy (HIPAA) and are available from the corresponding author upon reasonable request.

## Abbreviations

BMELs: Bone marrow edema-like lesions
BMI: Body mass index
FSE: Fast spin echo
ICCs: Intra-class correlation coefficients
IQR: Interquartile range
KL: Kellgren-Lawrence
MRI: Magnetic resonance imaging
OR: Odds ratio
PACS: Picture archiving and communication system
PD: Proton density
SE: Standard error
SD: Standard deviation
TE: Echo time
TR: Repetition time
VAS: Visual analogue scale
WORMS: Whole Organ MRI Scoring
